# Phenotypic expansion of *KCNH1* ‐associated disorders to include isolated epilepsy and its associations with genotypes and molecular sub‐regional locations

**DOI:** 10.1111/cns.14001

**Published:** 2022-10-25

**Authors:** Mao‐Qiang Tian, Ren‐Ke Li, Fan Yang, Xiao‐Mei Shu, Juan Li, Jing Chen, Long‐Ying Peng, Xiao‐Hua Yu, Chang‐Jian Yang

**Affiliations:** ^1^ Department of Pediatrics Affiliated Hospital of Zunyi Medical University Zunyi China; ^2^ Department of Pediatrics Guizhou Children's Hospital Zunyi China; ^3^ Cipher Gene LLC Beijing China

**Keywords:** epilepsy, *KCNH1* gene, status epilepticus, Temple‐Baraitser syndrome, Zimmermann‐Laband syndrome

## Abstract

**Purpose:**

Genotype‐phenotypic correlation of *KCNH1* variant remains elusive. This study aimed to expand the phenotypic spectrum of *KCNH1* and explore the correlations between epilepsy and molecular sub‐regional locations.

**Methods:**

We performed whole‐exome sequencing in a cohort of 98 patients with familiar febrile seizure (FS) or epilepsy with unexplained etiologies. The damaging effects of variants were predicted by protein modeling and multiple in silico tools. All reported patients with *KCNH1* pathogenic variants with detailed neurological phenotypes were analyzed to evaluate the genotype‐phenotype correlation.

**Results:**

Two novel *KCNH1* variants were identified in three cases, including two patients with FS with inherited variant (p.Ile113Thr) and one boy with epilepsy with de novo variant (p.Arg357Trp). Variant Ile113Thr was located within the eag domain, and variant p.Arg357Trp was located in transmembrane domain 4 of KCNH1, respectively. Two patients experienced refractory status epilepticus (SE), of which one patient died of acute encephalopathy induced by SE. Further analysis of 30 variants in 51 patients demonstrated that de novo variants were associated with epileptic encephalopathy, while mosaic/somatic or germline variants cause isolated epilepsy/FS. All hotspot variants associated with epileptic encephalopathy clustered in transmembrane domain (S4 and S6), while those with isolated epilepsy/seizures or TBS/ZLS without epilepsy were scattered in the *KCNH1*.

**Conclusions:**

We found two novel missense variants of *KCNH1* in three individuals with isolated FS/epilepsy. Variants in the *KCNH1* cause a spectrum of epileptic disorders ranging from a benign form of genetic isolated epilepsy/FS to intractable form of epileptic encephalopathy. The genotypes and variant locations help explaining the phenotypic variation of patients with *KCNH1* variant.

## Graphical abstract

Mutations in the *KCNH1* cause a spectrum of epileptic disorders ranging from a benign form of genetic isolated epilepsy/febrile seizure to intractable form of epileptic encephalopathy. The genotypes and variant locations help explaining the phenotypic variation of patients with *KCNH1* variant.

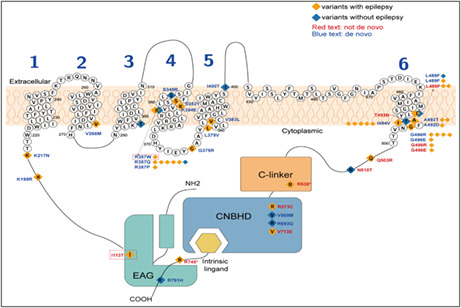

## 1 INTRODUCTION

*KCNH1* gene (OMIM* 603305), mapping to 1q32.2, encodes potassium voltage‐gated channel subfamily H member 1 (KCNH1). KCNH1 is a protein with 989 amino acids, containing an eag domain in N‐terminal, which is composed of a Per‐Arnt‐Sim (PAS) domain and a PAS‐cap domain, whereas the C‐terminal region contains a cyclic nucleotide‐binding homology domain (CNBHD), which is connected to the pore through a C‐linker domain. Besides, KCNH1 exhibits a typical Kv membrane topology with six transmembrane domains (S1–S6).^1^, ^2^ *KCNH1* is highly expressed in human brain, being essential for brain development (www.proteinatlas.org/ENSG00000143473‐KCNH1).^3^, ^4^ Clinically, variants in *KCNH1* have been associated with Temple‐Baraitser syndrome (TBS, OMIM# 611816) and Zimmermann‐Laband syndrome (ZLS, OMIM# 135500), two forms of neurodevelopmental disorder charactered by intellectual disability (ID), developmental disorder (DD), coarse face, gingival overgrowth, hypertrichosis, digital/toe anomalies, and seizures.^5^, ^6^, ^7^, ^8^, ^9^ Although epileptic seizures were usually observed in these syndromes, the genotype‐phenotypic associations of *KCNH1* are not fully understood, as pathogenic *KCNH1* variants have been identified in uncharacterized patients exhibited a part of the above phenotypes, including isolated epilepsy could be ascribed neither to a TBS nor to a ZLS. Here, we reported three cases harboring novel variants in the *KCNH1* gene suffering from epilepsy/febrile seizure (FS) and refractory status epilepticus (SE), but otherwise presenting distinct clinical features of TBS or ZLS, broadening the phenotypic spectrum of *KCNH1*. We also analyzed all previously reported patients with *KCNH1* variant, focusing on the correlations between epilepsy and molecular sub‐regional locations.

## 2 MATERIALS AND METHODS

### 2.1 Subjects

Patients with unexplained epilepsy or familial FS were recruited from Department of Pediatrics, Affiliated Hospital of Zunyi Medical University between July 2017 and March 2021. The studies adhered to the guidelines of the International Committee of Medical Journal Editors with regard to patient's consent for research or participation. This study was approved by the ethics committee of the Affiliated Hospital of Zunyi Medical University. We obtained written consents for genetic testing and publication of data for all patients.

Detailed clinical information of the patients was collected, including age, gender, epileptic types and frequencies, general and neurological examination results, family history, response to anti‐seizure medicines (ASMs), results of brain magnetic resonance imaging (MRI), and video‐electroencephalography (EEG). Epileptic seizures or epilepsies were diagnosed according to the criteria of the Commission on Classification and Terminology of the ILAE.^10^, ^11^, ^12^ FS is defined as seizures triggered by fever during aged 6 months to 5 years without a history of an unprovoked seizure or concurrent central nervous system infection.^13^, ^14^ SE is defined as convulsions persisting for >5 min. Refractory SE is defined as clinical or electroencephalographic seizures lasting >60 min despite treated with at least one first‐line ASMs (e.g., benzodiazepine) and one second‐line ASMs (e.g., phenytoin, phenobarbital, or valproate). Super‐refractory SE is defined as SE that has persisted or recurred for 24 h after the onset of general anesthesia treatment.^15^, ^16^, ^17^ Epilepsies with acquired causes were excluded.

### 2.2 Trios‐based WES

Blood samples were obtained from the probands and their parents to determine the origin of the identified genetic variants. Genomic DNA was extracted from peripheral blood using a QuickGene DNA whole blood kit (Fujifilm). Exome captures were performed using the IDT xGen Exome Research Panel with paired‐end read sequences generated on NovaSeq 6000 sequencing. Sequences were aligned to Human reference genome GRCh38/hg38. The variants were then annotated through AnnoVar^18^ and evaluated according to allele frequencies, pathogenicity prediction, and protein function. Pathogenic variants related to clinical phenotypes will further be verified by Sanger sequencing.

### 2.3 Mutation analysis

Aiming to evaluate the genotype‐phenotype correlation, we exhaustively searched *KCNH1* pathogenic variants on the PubMed up until Mar 2022 to identify studies published in English using the following terms: KCNH1, epilepsy, seizure, TBS, ZLS, Temple‐Baraitser, Zimmermann‐Laband. All pathogenic variants in patients with detailed neurological phenotypes were analyzed.

Molecular modeling analysis was performed to show the variations in protein structure. The human_KCNH1 model was downloaded in the AlphaFold dataset.^19^ UCSF Chimera software was used for three‐dimensional protein structure visualization and analysis. DUTE server (http://biosig.unimelb.edu.au/duet/) and Grantham scores^20^ were used for prediction of protein stability changes. The changes of the protein stability were assessed using the free energy stability change (DDG, kcal/mol) value.

## 3 RESULTS

### 3.1 Identification of *KCNH1* variants

**FIGURE 1:**
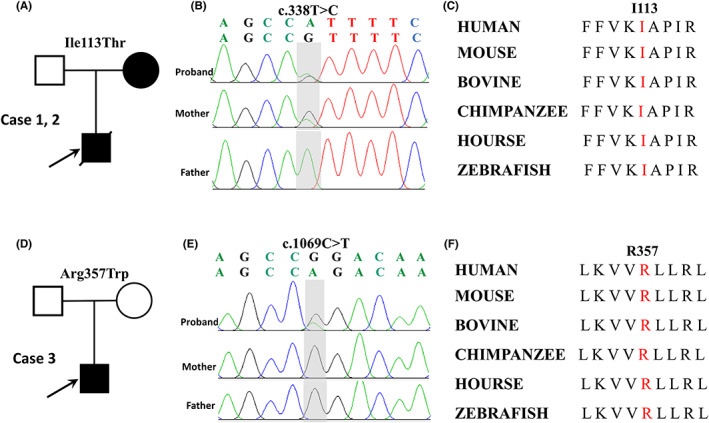
Genetic data on the patients with *KCNH1* variants. (A, D) Pedigree of the two families. The filled symbol with arrows identifies the probands. (B, E) The variants c.338T>C and c.1069C>T were identified through whole‐exome sequencing and confirmed by Sanger sequencing. The SNVs were shown in gray shadows, c.338T>C was inherited from his mother, and c.1069C>T was de novo. (C, F) The variant amino acids in our patient were conserved from multiple species.

A total of 98 patients were recruited; among them, two missense *KCNH1* variants were identified in three cases (Table 1; Figure 1A,D), including one inherited variant (c.338T>C; p.Ile113Thr) and one de novo variant (c.1069C>T; p.Arg357Trp). The three cases had no other pathogenic or likely pathogenic variants. Two variants of *KCNH1* were annotated based on the transcript NM_172362 and confirmed by Sanger sequencing (Figure 1B,E).

**TABLE 1:** Clinical features of the individuals with *KCNH1* variants

	Variants (NM_172362)	Gender	Age at report	Diagnosis	Age of onset	Seizure & frequency	ASM	ID/DD	EEG	Brain MRI	Outcomes
1	c.338T>C; p.Ile113Thr	Male	4 yrs	FS, supper refractory SE	1 yrs	3–4 simple FS, febrile supper refractory SE at age of 4, GTCS	–	No	Severe abnormal. See Figure 2A	Abnormal at age of 4 (see Figure 2B)	Died due supper refractory SE and brain damage
2	c.338T>C; p.Ile113Thr	Female	32 yrs	FS,	1.5 yrs	4–5 times FS, GTCS	–	No	NA	NA	Seizure‐free after age of 6 yrs
3	c.1069C>T; p.Arg357Trp	Male	2 yrs	Dravet syndrome, refractory SE	8 months	PE or generalized, 10+ times triggered by fever /hot water bath, SE	VPA	Mild DD	Abnormal. See Figure 2C	Normal	Seizure‐free for 10 months

Abbreviations: ASM, anti‐seizure medicine; DD, developmental disorder; EEG, electroencephalogram; FS, febrile seizure; GTCS, generalized tonic‐clonic seizure; ID, intellectual disability; MRI, magnetic resonance imaging; NA, not available; SE, status epilepticus; VPA, valproate; yr, year.

The amino acid residues of the two missense variants are highly conserved in various species (Figure 1C,F). The two missense variants were suggested to be damaging/disease‐causing/conserved by at least three silico tools (Table 2). The two variants are not present in gnomAD database (Table 2). Variant p.Arg357Trp was predicted to have more severe effects than variant p.Ile113Thr (101 vs. 89) according to Grantham scores.

**TABLE 2:** Genetic features of the individuals with *KCNH1* variants

Cases	Position	cDNA change (NM_172362)	Protein change	MAF	MAF‐EAS	Mutation taster	SIFT	CADD	Polyphen2_HDIV	GERP++	PhyloP	Grantham scores
1, 2	Chr1:211090663	338T>C	p.Ile113Thr	–	–	DC (1.00)	D (0.09)	D (26.30)	PD (0.99)	C (5.22)	C (8.93)	89
3	Chr1:210920033	1069C>T	p.Arg357Trp	–	–	DC (1.00)	D (0.00)	D (34.00)	PD (1.00)	C (5.48)	C (4.26)	101

Abbreviations: B, benign; C, conserved; CADD, combined annotation dependent depletion; D, damaging; DC, disease‐causing; Chr, chromosome; MAF, minor allele frequency from Genome Aggregation Database; MAF‐EAS, minor allele frequency from East Asia population in Genome Aggregation Database; NA: not applicable; PD, probably damaging.

### 3.2 Clinical information

The three patients showed infancy or childhood‐onset seizures (8 months–1.5 years). The main clinical features of the cases are summarized in Table 1. Three patients were all born to non‐consanguineous parents after an uneventful pregnancy.

**FIGURE 2:**
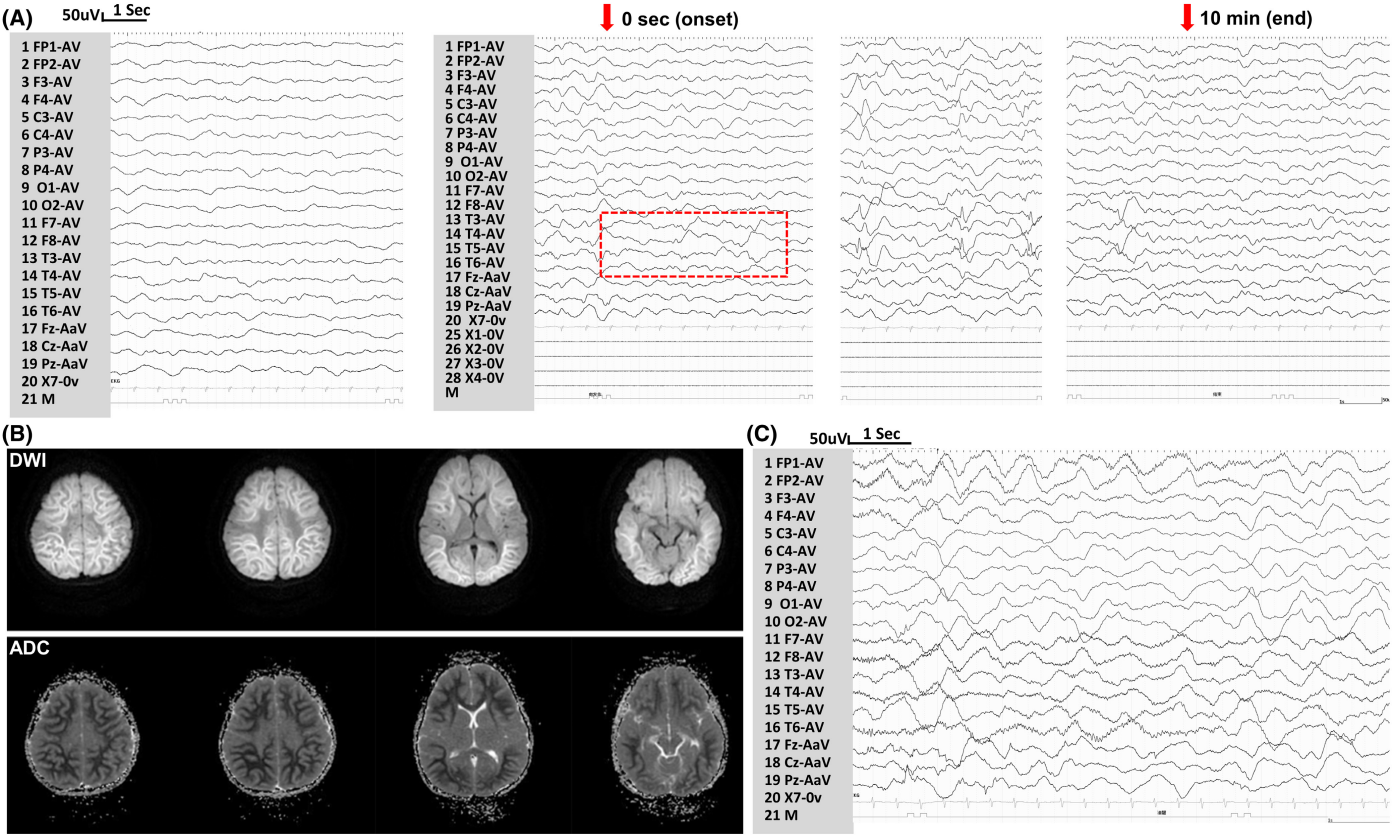
EEG and neuroimaging results in the cases with *KCNH1* variants. (A) EEG of case 1 detected diffuse slow waves, and subclinical seizures originated from bilateral temporal (red box indexed the seizure onset). (B) Brain MRI showed bright tree appearance in DWI, and low‐intense signal in ADC. (C) EEG of case 3 indicated diffuse slow waves and drug‐related fast waves. (DWI, diffuse weighted images; ADC, apparent diffusion coefficient)

Case 1 and case 2 harbored variant p.Ile113Thr. Case 1 was a 4‐year‐old boy. He developed simple FS at frequency of 1–2 times yearly since age of 1 year. Psychomotor development was normal. EEG and brain MRI were unremarkable. He experienced febrile‐induced SE at age of 4, his seizures, which lasted for 1 h, ceased after the administration of intravenous continuous infusion of valproate (1.5 mg/kg/h). He was comatose, despite the disappearance of the seizures. His Glasgow Coma Scale score was 6/15 (E2 + V2 + M2). He developed refractory SE 2 days after admission, as his seizures were unresponsive to standard use of diazepam, intravenous valproate, and phenobarbital. Super‐refractory SE was diagnosed because seizures remained uncontrolled 24 h after initiating continuous intravenous use of midazolam and propofol. No dysmorphic features were observed. Except for obvious intracranial hypertension (310 mmH_2_O), no abnormality was found in cerebrospinal fluid tests. Routine blood testing results were unremarkable. EEG revealed diffuse slow waves, and subclinical seizures lasted for 10 min originated from bilateral temporal were present (Figure 2A). Brain MRI (5th day of seizure onset) showed bilateral hemisphere cerebral edema characterized by diffuse subcortical white matter lesions. Bright tree appearance (subcortical white matter hyper‐intense signal) in diffuse weighted images and low‐intense signal in apparent diffusion coefficient were observed on MRI (Figure 2B). Subsequently, diagnosis of acute encephalopathy after SE was made based on his clinical and neuroimaging features. Despite aggressive therapeutic strategies were given, the patient eventually died 20 days after this seizure onset due to uncontrollable seizures and severe brain damage.

Case 2, the mother of case 1, is currently 32 years old. She had 4–5 times of FS since the age of 1.5, and she did not have seizure after the age of 5. Neither neuroimaging nor EEG were performed. Her neuropsychology is normal at present.

Variant p.Arg357Trp was identified in case 3. This boy experienced FS (generalized or focal) since age of 8 months. Subsequently, he experienced frequently seizures triggered by low‐grade fever or hot‐water bath, which led to a diagnosis of Dravet syndrome. Brain MRI and EEG were normal at age of 1 year. At age of 1 year and 2 months, he had short‐duration but frequent (>10 times/h) seizures triggered by fever, which were resistant to multiple ASMs, including diazepam, valproate, phenobarbital. Subsequently, his seizures stopped until continuous intravenous infusion of midazolam (0.24 mg/kg/h). EEG showed diffuse slow waves and drug‐related fast waves (Figure 2C). He became seizure‐free with treatment of valproate (22 mg/kg/day) till the last follow‐up at age of 2 years even when the body temperature was as high as 40°C. Mild developmental delay was observed. He can walk without support at age of 1 year and 7 months and can speak 3–4 words at age of 2 years. Gesell Developmental Observation‐Revised screening was performed, and the results showed a mild delay in gross motor development, language, and social‐emotional responses. No any dysmorphic feature or malformations was observed, including hypoplasia of the nails, coarse face (Figure S1).

### 3.3 Structural alteration of KCNH1 protein

**FIGURE 3:**
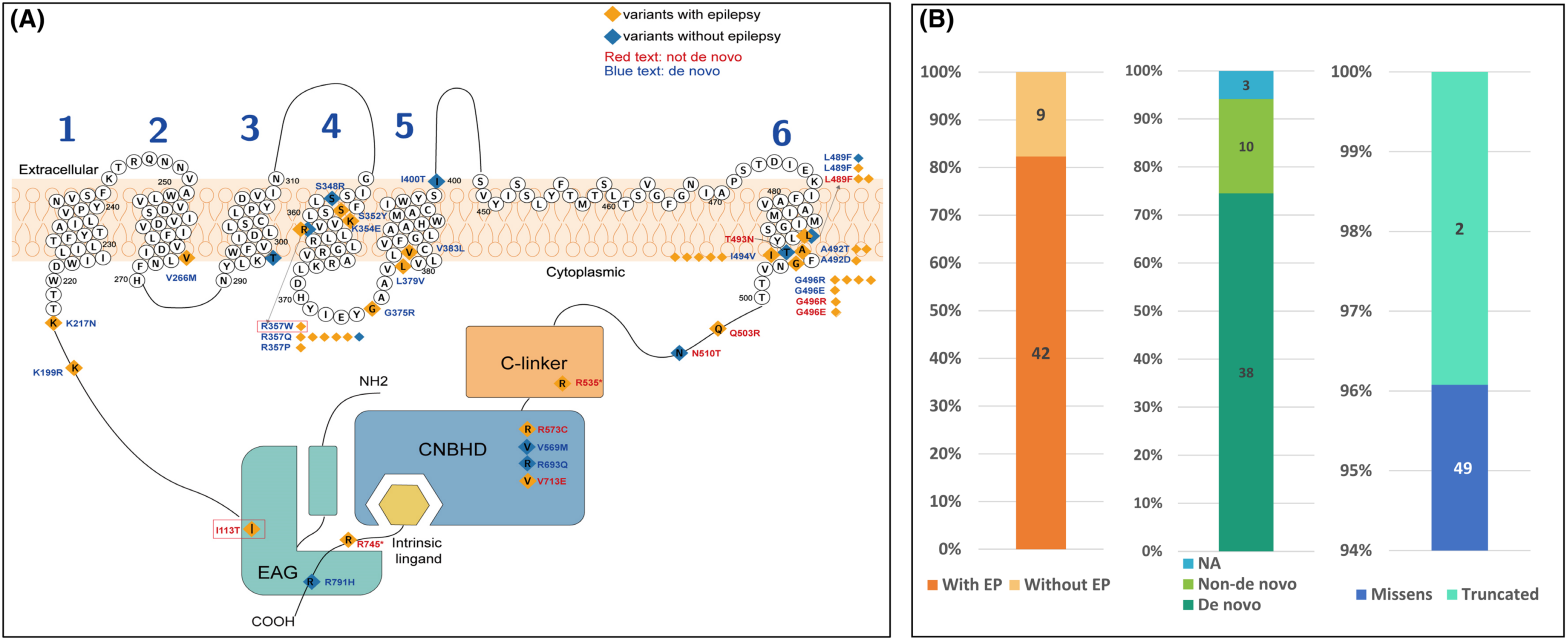
Schematic diagram of variant sites and phenotypes/genotypes characteristics of the patients reported to date. (A) Schematic diagram of the transmembrane structure. The CNBHD, C‐linker, and EAG domains were shown in the picture. The S1–S4 segments act as voltage‐sensor domains. The KCNH1 channel shows the location of the residues affected in individuals with (orange square) or without (blue square) epilepsy. Most of them were de novo variants (blue text), and few were inherited or not known (red text). Variants in our patients were highlighted with red boxes. (B) Showed the clinical phenotypes, and genetic characteristics of the patients reported to date

As shown schematically in Figure 3A, KCNH1 contains eag domain and CNBHD located in cytoplasmic and six transmembrane domains (S1–S6). Structural model of KCNH1 indicated variant p.Ile113Thr was located within the eag domain, and variant p.Arg357Trp was located in S4.

**FIGURE 4:**
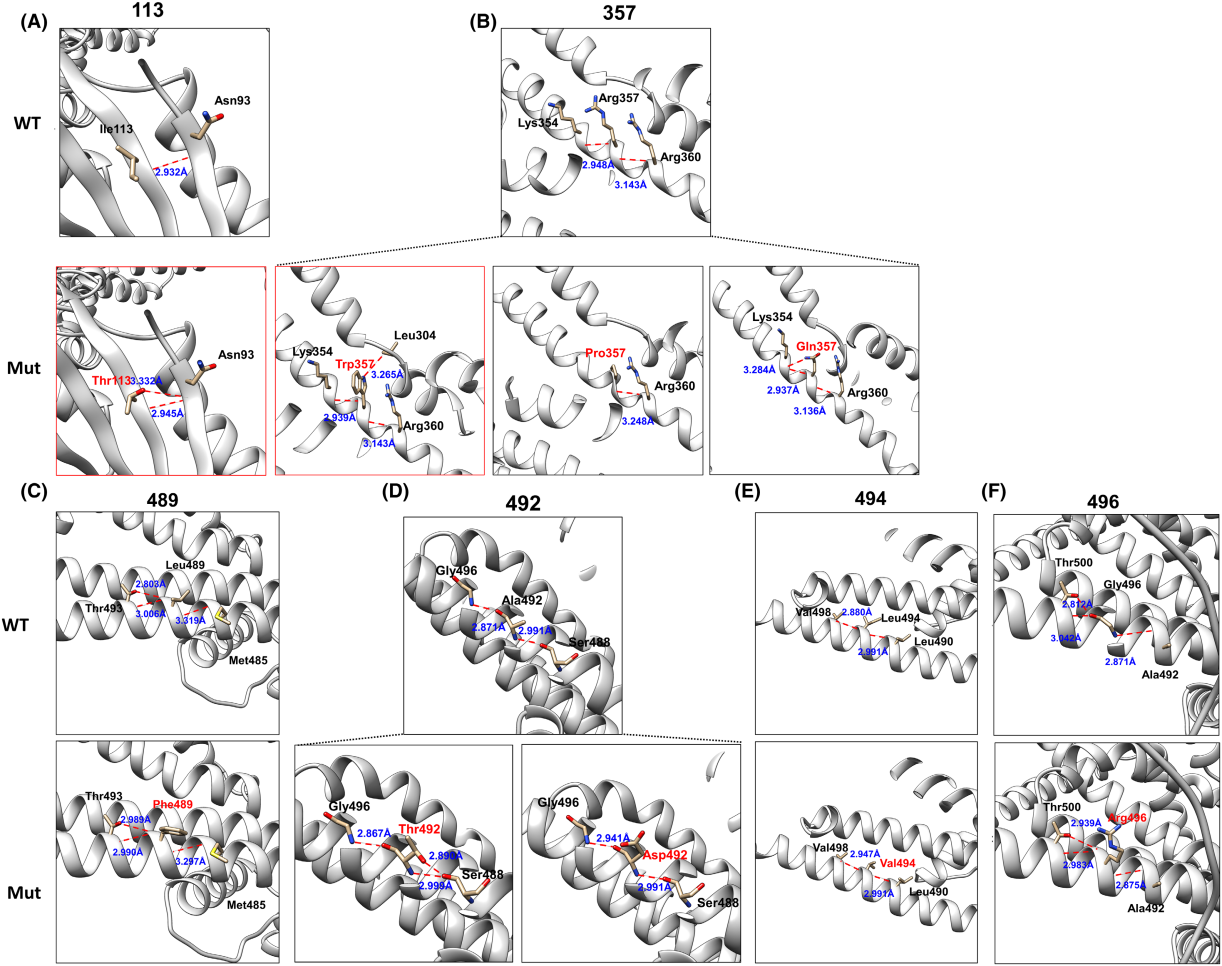
Changes in KCNH1 protein structure. (A–F) The protein changes including our variants and five hotspots (p.Arg357, p.Leu489, p.Ala492, p.Leu494, and p.Gly496). The wild‐type protein structures were shown in the top row, and the mutant structures were in the row below. Hydrogen bonds are represented by red dashed lines and the distances are highlighted with blue text. Structures in our patients were highlighted with red boxes.

DUET server was used to analyze the effects of missense variants on protein stability. Results showed destabilizing for the residues' changes. Variant p.Ile113Thr and p.Arg357Trp were predicted to be least stable with DDG value of −3.159 and −0.448 kcal/mol, respectively. Both variants changed the hydrogen bonds (Figure 4A,B). Residue Ile113 originally formed one hydrogen bond with residue Asn93. The missense variant p.Ile113Thr results in an additional hydrogen bond with residue Asn93. Residue Arg357 originally formed hydrogen bonds with residue Lys354 and residue Arg 360, respectively. When arginine was replaced by tryptophan at residue Arg357, a new hydrogen bond with residue Leu304 was formed. We also analyzed the previous reported five reported hotspot variants (p.Arg357, p.Leu489, p.Ala492, p.Leu494, and p.Gly496) and found that all variants changed numbers or distances of hydrogen bonds (Figure 4B–F).

### 3.4 Genotype‐phenotype correlation of *KCNH1* variants

We analyzed genotype‐phenotypic associations in all reported *KCNH1* pathogenic variants with detailed neurological phenotypes. Previously, 28 *KCNH1* variants in 48 patients have been reported,^4^, ^5^, ^9^, ^21^, ^22^, ^23^, ^24^, ^25^, ^26^, ^27^, ^28^, ^29^, ^30^, ^31^, ^32^, ^33^, ^34^, ^35^, ^36^, ^37^ including the present data, a total of 30 variants in 51 patients. Forty‐two out of 51 patients had epilepsy. Thirty‐eight patients harbored de novo variants, 13 patients harbored non‐de novo variant (inherited, mosaic or unknown origin; Figure 3B). Forty‐nine out of 51 patients harbored missense variants. Among the only two nonsense variants,^34^, ^37^ variant p.R535* was also found in clinically unaffected father and sister; the patient carrying variant p.R745* also harbored another epileptic encephalopathy‐related, pathogenic variant, *CACNA1A* (c.2134G>A; p.Ala712Thr), this variant has been reported as a pathogenic variant in epileptic encephalopathy patients.^38^ Clinical and molecular details of patients with epilepsy are listed in Table 3, and patients without epilepsy were listed in Table S1.

**TABLE 3:** Patients with *KCNH1* variant with epilepsy

References	Age/sex	Variants (NM_172362)	Inheritance	Onset of seizures	Epilepsy/seizure types	TBS/ZLS	Intellectual disability (ID)/developmental delay (DD)	Effective ASM and outcomes of epilepsy
De novo								
vonWrede^37^	6 yrs 4 mons/M	c.596A>G; p.Lys199Arg	De novo	2.5 yrs	FS, DEE, GTCS, myoclonic, SE	No	Psychomotor development was severely impaired	Cortisone, perampanel, and topiramate were effective
Simons^33^	6 yrs/F	c.651G>C; p.Lys217Asn	De novo	6 yrs	Generalized tonic‐clonic seizure	TBS	Mild psychomotor development delay	NA
Rochtus^29^	11 yrs/M	c.796G>A; p.Val266Met	De novo	2.5 yrs	Myoclonic, GTCS, absence, and drop seizures	No	Mild delay	NA, but she got seizure‐free and off medication
Kortüm^25^	21 yrs/M	c.1055C>A; p.Ser352Tyr	De novo	Childhood	Frequent seizures, SE	ZLS	Profound ID/DD	NA
Gripp^24^	2.9 yrs/F	c.1060A>G; p.Lys354Glu	De novo	4 weeks	NA	TBS	Motor and speech delay; skills at 4 m level at age 2.8	NA
Present study	1 yrs 10 mons/M	c.1069C>T; p.Arg357Trp	De novo	8 mons	Fever sensitivity, GTCS, focal, SE.	No	Mild delay	Valproic acid, seizure‐free
Fukai^23^	8 yrs/M	c.1070G>A; p.Arg357Gln	De novo	6 mons	generalized tonic‐clonic	TBS	Severe ID and DD	Epilepsy was controlled by valproic acid and zonisamide
Bramswig^5^	14 yrs/F	c.1070G>A; p.Arg357Gln	De novo	1.5 yrs	Focal epilepsy with secondary generalization	TBS	Severe ID and DD	NA
Bramswig^5^	4 yrs 4 mons/F	c.1070G>A; p.Arg357Gln	De novo	2 yrs 9mons	Frequent temporal epilepsy, SE	TBS	Severe ID and DD, ASD	NA
Fukai^23^	6 yrs/M	c.1070G>A; p.Arg357Gln	De novo	1 mon	Focal seizures	No	Severe ID and DD	His seizures were controlled by frisium, trileptin, and risperdal
Gripp^24^	9.7 yrs/F	c.1070G>A; p.Arg357Gln	De novo	2.75 yrs	Focal, GTCS, and absences	TBS	Severe DD/ID	NA
Fukai^23^	7 yrs/M	c.1070G>C; p.Arg357Pro	De novo	4 mons	Generalized tonic‐clonic	No	Severe ID and DD	Carbamazepine, and clobazam, relative effectiveness
Kortüm^25^	19 yrs/F	c.1123G>A; p.Gly375Arg	De novo	Adolescence	NA	ZLS	Severe/profound ID	NA
Kortüm^25^	7 yrs/M	c.1135C>G; p.Leu379Val	De novo	8 mons	Focal clonic secondary generalized seizures, SE	ZLS	Severe intellectual and motor disability	Generalized drug‐resistant seizures
Kortüm^25^	21 yrs/M	c.1147G>C; p.Val383Leu	De novo	Childhood	Frequent seizures,	ZLS	Profound DD/ID	NA
Bramswig^5^	14 yrs/F	c.1465C>T; p.Leu489Phe	De novo	9 mons	GTCS, absences	TBS	Severe ID and DD	Seizures were responding well to lamotrigine and ethymal
Rochtus^29^	3 yrs/M	c.1474G>A; p.Ala492Thr	De novo	Neonatal	DEE	No	Severe ID and DD	NA
Froukh^22^	5 mons/M	c.1474G>A; p.Ala492Thr	De novo	NA	DEE	TBS	Mild DD/ID	NA
Mastrangelo^28^	NA	c.1475C>A; p.Ala492As	De novo	Infancy	GTCS	No	Severe ID/DD	Responsive to levetiracetam
Simons^33^	3 yrs 7 mons/M	c.1480A>G; p.Ile494Val	De novo	3 yrs	FS, GTCS	TBS	Moderate ID/DD	NA
Simons^33^	4 yrs/M	c.1480A>G; p.Ile494Val	De novo	4 yrs	NA	TBS	Severe ID/DD	NA
Simons^33^	9 yrs/F	c.1480A>G; p.Ile494Val	De novo	8 yrs	NA	TBS	Severe ID/DD	NA
Kortüm^25^	12 yrs 8 mons/F	c.1480A>G; p.Ile494Val	De novo	10 yrs 6 mons	Generalized tonic‐clonic seizures	TBS	Severe DD/ID, autism	NA
Kortüm^25^	4 yrs 6 mons/F	c.1480A>G, p.Ile494Val	De novo	Neonatal	Focal motor seizure, SE	TBS	Severe DD/ID	Seizures were responsive to phenytoin, valproic acid
Kortüm^25^	12 yrs/F	c.1486G>A; p.Gly496Arg	De novo	6 mons	Focal epilepsy	TBS	Moderate DD/ID	Controlled by tegretol
Mastrangelo^28^	15 yrs/F	c.1486G>A; p.Gly496Arg	De novo	Infancy	Tonic, tonic‐clonic, 2 SE	No	Severe ID	Responsive to lamotrigine, carbamazepine and clobazam
Mastrangelo^28^	9 yrs/F	c.1486G>A; p.Gly496Arg	De novo	Infancy	Focal clonic, generalized tonic‐clonic, myoclonic	No	Mild/moderate ID/DD	Responsive to carbamazepine
Mastrangelo^28^	1.7 yrs/F	c.1486G>A; p.Gly496Arg	De novo	Infancy	Generalized tonic‐clonic, focal clonic, myoclonic	No	Mild ID	Responsive to levetiracetam
Fukai^23^	3 yrs/M	c.1487G>A; p.Gly496Glu	De novo	Neonatal	Generalized convulsions	No	Severe DD and ID	Valproate, clobazam, and levetiracetam were effective
Inherited or mosaic								
Present study	32 yrs/F	c.338T>C; p.Ile113Thr	Parents not tested	Childhood	GTCS	No	No	Medicine was not given, seizure‐free
Present study	4 yrs/M	c.338T>C; p.Ile113Thr	Maternal	1.5 yrs	GTCS, super SE	No	No	Medication was not given, dead due to SE
Simons^33^	NA/F *a*	c. 1465C>T; p.Leu489Phe	Mosaic (blood 13.6%)	NA	NA	No	No	NA
Simons^33^	14 yrs/F *b*	c. 1465C>T; p.Leu489Phe	Maternal?	4 yrs	NA	TBS	Mild/moderate DD/ID	Controlled by topiramate and carbamazepine
Simons^33^	NA/F c	c.1508A>G; p.Gln503Arg	Mosaic (blood 4.7%; saliva 4.5%; fibroblast 3.4%)	Childhood	Generalized seizure	No	No	Epilepsy was well controlled with carbamazepine.
Simons^33^	4 yrs/M *d*	c.1508A>G; p.Gln503Arg	Maternal?	2 yrs	Generalized seizure	TBS	Mild ID/DD	Well controlled on phenytoin
vonWrede^37^	36 yrs/F	c.1603C>T; p.Arg535*	Paternal	2 yrs	GTCS, atonic, focal seizures	No	Mild/moderate ID/DD	Controlled by sultiame, phenytoin, and clonazepam
Rochtus^29^	6 yrs/M	c.1717C>T; p.Arg573Cys	Paternal	2 mons	Focal seizures	No	Global developmental delay	NA
vonWrede^37^	40 yrs/F	c.2138T>A; p.Val713Glu	Maternal	13 yrs	Absence, tonic, GTCS)	No	No	Drug‐resistant, cannabidiol was partial effective
vonWrede^37^	40 yrs/M	c.2138T>A; p.Val713Glu	Mosaic (brain tissue)	5 yrs	Multiple seizures, FCD IIb	No	No	Drug‐resistant epilepsy
Unknown origin								
Gripp^24^	34 yrs/F	c.1486G>A; p.Gly496Arg	Father not tested	Neonatal	GTCS, several times SE	ZLS	Mild DD, moderate ID	NA
Gripp^24^	39 yrs/F	c.1487G>A; p.Gly496Glu	Parents not tested	Prenatal	Myoclonic jerks, mixed seizure type	TBS	Severe DD and ID	NA, improved but not stopped by medication
Takata^34^	NA	c.2233C>T; p.Arg745*	NA	NA	DEE	NA	Developmental encephalopathy	NA

Abbreviations: DEE, developmental epileptic encephalopathy; F, female; FS, febrile seizure; GTCS, generalized tonic‐clonic seizure; ID/DD, intellectual disability/developmental disorder; M, male; mon, month; NA, not available; SE, status epilepticus; TBS, Temple‐Baraitser syndrome; ZLS, Zimmermann‐Laband syndrome.

We further analyzed the sub‐regional locations of all variants demonstrating that 83% (35/42) variants in patients with epilepsy/seizures were located in the transmembrane domains. De novo variants associated with epilepsy have obvious spatial clustering properties, five hotspot/recurrent variants including p.Arg357 (eight patients), p.Leu489 (four patients), p.Ala492 (three patients), p.Leu494 (five patients), and p.Gly496 (seven patients) were observed and all located in the transmembrane domains. Except for p.Arg357 located in S4, all other hotspot variants are located in S6 (Figure 3A).

Among the 42 patients with epilepsy, more than half of patients (58%, 20/34) were pharmaco‐responsive, of which 10 patients became seizure‐free. 21% (9/42) of patients had SE. We also found that patients with inherited or mosaic/somatic variants have a more mild phenotypes than patients with de novo variants, specifically: later age of seizure onset (2.1 vs. 2.7 years of age), fewer incidences of ID/DD (40%, 4/10 vs. 100%, 41/41), and higher rate of seizure‐free (63%, 5/8 vs. 19%, 5/26).

## 4 DISCUSSION

Present study provided a clinical description of three individuals with two novel missense variants of *KCNH1* with FS/epilepsy and refractory SE without features of TBS/ZLS. One patient had mild ID with drug‐responsive Dravet syndrome and finally got seizure‐free. In the familial cases of FS, one patient died of super refractory SE. Both variants had no allele frequency in the gnomAD. The two variants affected residues conserved through evolution and invariantly observed among vertebrates. The two variants were predicted to be damaging by multiple in silico tools and altered the protein conformation. Taking together the evidence that *KCNH1* gene is predominantly expressed in brain and associated with neurodevelopment and neural excitability,^3^, ^4^ the two variants of *KCNH1* were suggested to be the pathogenic gene of the current cases.

We analyzed the largest cohort of 51 patients with *KCNH1* variants to date and found that epilepsy/seizures were present in 82% individuals suggesting a direct role of *KCNH1* in epileptogenesis. However, the seizure types, severity, and response to ASMs varied widely. Patients with inherited or mosaic/somatic variants have milder phenotypes than patients with de novo variants, including later age of seizure onset, fewer incidences of ID/DD, and higher rate of seizure‐free. Of the 10 patients with hereditary or mosaic/somatic variant, most of them presented with isolated epilepsy without TBS/ZLS. These findings provided possible evidence that a low level of mosaic/somatic variant or a weaker effect on KCNH1 function of inherited variant may contribute to isolated epilepsy phenotype.

In addition to inheritance and variant patterns that determine phenotypic differences, recent studies have showed that molecular sub‐regional location of variants was also a critical factor to determine the pathogenicity of variants and associated with phenotypic variations.^39^, ^40^, ^41^ In this study, all patients with hotspot variants (p.Arg357, p.Leu489, p.Ala492, p.Ile494, and p.Gly496) associated with epilepsy with moderate to severe ID/DD clustered in S4 and S6, while those with isolated epilepsy/seizures or TBS/ZLS without epilepsy were scattered, suggesting a molecular sub‐regional effect of *KCNH1* variants. One of our newly reported patients presented severe phenotype of Dravet syndrome had a variant (p.Arg357Trp) in S4. Another patient had variant (p.Ile113Thr) in the eag domain near N‐terminal; this patient showed a mild phenotype‐FS. These results demonstrate the important role of voltage‐sensing transmembrane helix S4 and S6 of the KCNH1 channel in maintaining neuronal excitability and development.

However, factors influencing clinical phenotypic heterogeneity of patients with *KCNH1* variants remain not fully elucidated, because we found that even patients harbored variants located in S6, their clinical presentations varied widely. Fourteen patients with variants at p.Leu489, p.Ala492, and p.Gly496; all showed early onset of epilepsy within 2 years of age, while five patients with p.Ile494Val variant had late‐onset of epilepsy, with an average age of 5. We found variant Ile494Val appears to have the least effect on hydrogen bonds or distances of inter‐amino acid, which may help explain the mild phenotype of patients with p.Ile494Val variant. However, the specific mechanism is currently unknown; further accumulation of cases is needed for detailed research on effect of *KCNH1* variant on spatial structure of KCNH1.

When comparing de novo variants in patients with neurodevelopmental disorders, individuals with missense variants have been reported to be generally more likely to develop epilepsy than individuals with truncating variants, and enrichment was observed in genes associated with ion channel‐encoding genes (*KCNQ2*, *SCN1A*, and *KCNH1*),^42^, ^43^, ^44^ suggesting a dominant‐negative or gain‐of‐function effect of *KCNH1* variant in the pathophysiology of epilepsy. In *KCNH1*, studies in both Xenopus laevis oocytes and human HEK293T cells have revealed that missense variants lead to deleterious gain‐of‐function effect.^2^, ^25^, ^33^ Present study further supports this view, as 96% of patients harbored missense variants. There are evidences of non‐pathogenicity for only two truncated variants: variant p.Arg535* had very low penetrance, as two healthy individuals in the family also carried this variant; patient with variant p.Arg745* also carried another pathogenic epileptic encephalopathy‐related gene. These findings indicated that gain‐of‐function effect is the pathogenic mechanism of *KCNH1* variant, because variants lead to haploinsufficiency in this gene appear to be better tolerated.

In this study, more than half of patients with epilepsy (20/34) responded well to the ASMs. However, these patients are prone to develop SE, because two of our newly reported patients developed super refractory SE, and 21% of the reported patients experienced SE. SE may induce acute encephalopathy which is characterized by non‐inflammatory encephalopathy, followed by prolonged consciousness disturbance, and often followed by severe neurological sequelae.^45^, ^46^ As occurred in our case 1, patients with *KCNH1* variant may also develop acute encephalopathy after SE like patients with other ion channel‐encoding genes variant, such as *KCNQ2* and *SCN1A*.^46^

This study has several limitations. The direct functional effects of the variants were not examined. More cases are needed to elucidate genotype‐phenotype correlation of *KCNH1* gene in future studies.

## 5 CONCLUSIONS

In conclusion, we found two novel missense variants of *KCNH1* in three individuals with FS/epilepsy. Variants in the *KCNH1* cause a spectrum of epileptic disorders ranging from benign isolated epilepsy/FS to severe epileptic encephalopathy. Low doses of mosaic/somatic variants and less deleterious germline variants may cause isolated epilepsy/FS, while de novo gain‐of‐function variants always cause developmental syndromic disorders with or without epilepsy. The genotypes and variant locations help explain the phenotypic heterogeneity of patients with *KCNH1* variant.

## AUTHOR CONTRIBUTIONS

Mao‐Qiang Tian, Ren‐Ke Li, and Xiao‐Mei Shu collected the data from patients, reviewed the literatures, and wrote the paper; Juan Li analyzed genetic pathogenicity; Jing Chen and Fan Yang performed whole exome sequencing data analysis; Long‐Ying Peng, Xiao‐Hua Yu, and Chang‐Jian Yang analyzed EEG recordings and neuroimaging data.

## FUNDING INFORMATION

This work was supported by grants from Basic Research Program of Guizhou Province: Guizhou Science and Technology Foundation (Grant No. ZK [2021] General 418).

## CONFLICT OF INTEREST

All authors claim that there are no conflicts of interest.

## INFORMED CONSENT

The patients gave their informed consents for this report.

## Supporting information


Figure S1

Appendix S1

Table S1

## Data Availability

The data that support the findings of this study will be available from the corresponding author upon reasonable request.
