# Supplementary figures and images for: Phenotypic expansion of KCNH1 ‐associated disorders to include isolated epilepsy and its associations with genotypes and molecular sub‐regional locations
Source: CNS Neurosci Ther. 2022 Oct 25;29(1):270–81. doi: 10.1111/cns.14001 (PMC9804083; doi:10.1111/cns.14001)

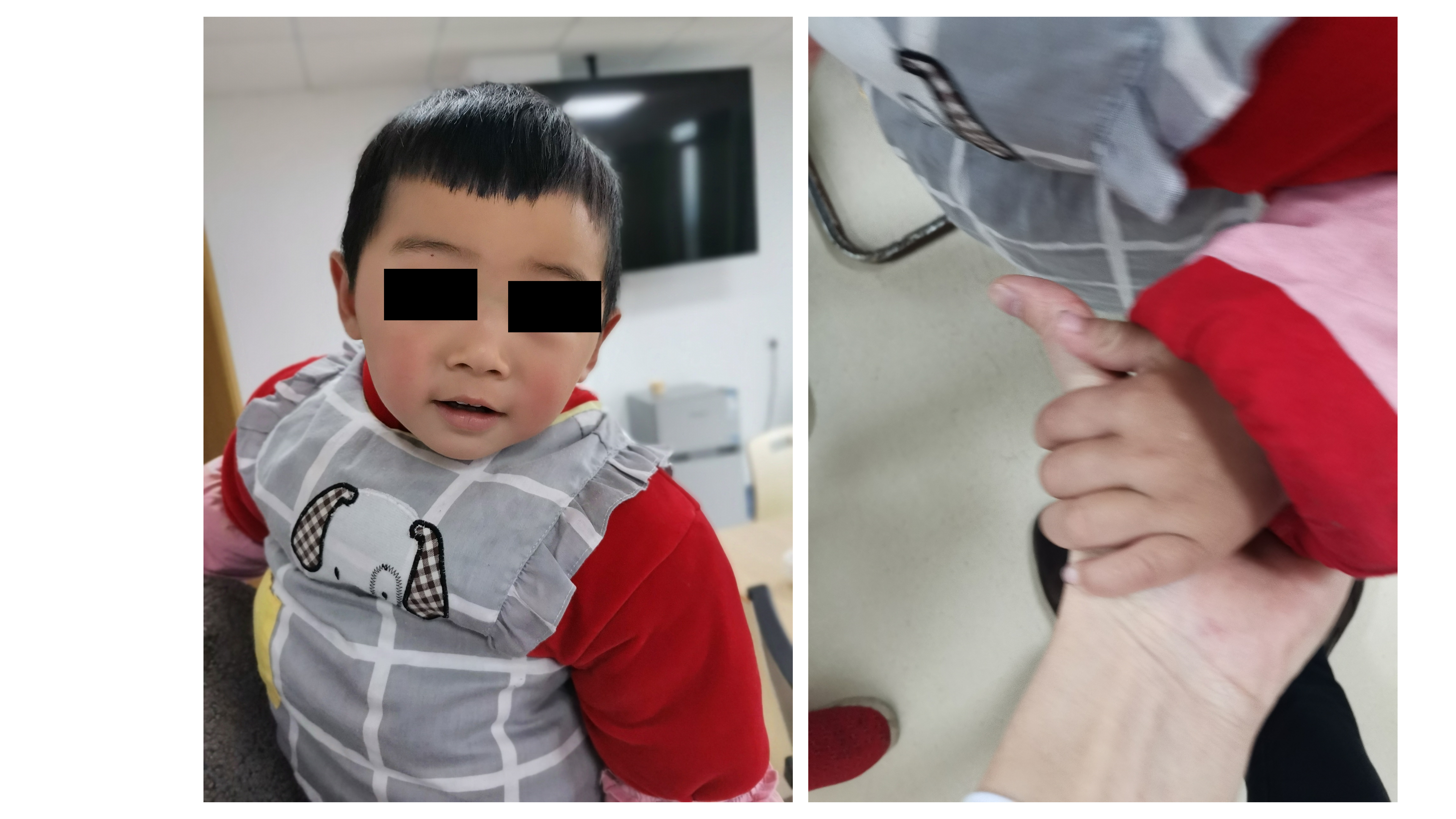

Supplement: Supplementary file 1 — Figure S1 [file CNS-29-270-s002.tif]
