# Supplementary material for: Phenotypic expansion of KCNH1 ‐associated disorders to include isolated epilepsy and its associations with genotypes and molecular sub‐regional locations
Source: CNS Neurosci Ther. 2022 Oct 25;29(1):270–81. doi: 10.1111/cns.14001 (PMC9804083; doi:10.1111/cns.14001)
Supplement: Supplementary file 2 — Appendix S1 [file CNS-29-270-s003.docx]

Supplementary figure S1 is: No any dysmorphic features or malformations were observed for case 3 till the last follow-up at age of 2 years.
