# Supplementary material for: Phenotypic expansion of KCNH1 ‐associated disorders to include isolated epilepsy and its associations with genotypes and molecular sub‐regional locations
Source: CNS Neurosci Ther. 2022 Oct 25;29(1):270–81. doi: 10.1111/cns.14001 (PMC9804083; doi:10.1111/cns.14001)
Supplement: Supplementary file 3 — Table S1 [file CNS-29-270-s001.docx]

**Table S 1,** Patients with *KCNH1* mutation without epilepsy

|  | Age | Mutation | Inheritance | Phenotype | [Intellectual disability](javascript:;) (ID)/ Developmental delay (DD) |
| --- | --- | --- | --- | --- | --- |
| Mastrangelo(1) | 28 yrs/F | c.881C>T; p.Thr294Met | De novo | No | severe ID |
| Mégarbané(2) | 15 mons/M | c.1042G > A; p.Gly348Arg | De novo | TBS | severe ID |
| Bramswig(3) | 4 years and 4 mon/M | c.1070G>A; p.Arg357Gln | De novo | TBS | severe ID |
| Satterstrom; Cappi(4, 5) | NA | c.1199T>C; p.Ile400Thr | De novo | ASD | No |
| Gripp (6) | 14 mons/M | c.1465C>T; p.Leu489Phe | De novo | TBS | Severe ID/DD |
| Mastrangelo(1) | 3.5 yrs/F | c.1478C>A; p.Thr493Asn | De novo | No | moderate/severe ID |
| Wang(7) | NA | c.1529A>C; p. Asn510Thr | NA | TBS | NA |
| Rossi(8) | NA | c.1705G>A; p. Val569Met | De novo | TBS, ASD | ID |
| Mastrangelo(1) | 7.9 yrs/M | c.2078G>A; p.Arg693Gln | De novo | No | severe ID |

References

1. Mastrangelo M, Scheffer IE, Bramswig NC, Nair LD, Myers CT, Dentici ML, et al. Epilepsy in KCNH1-related syndromes. Epileptic Disord. 2016 Jun 1;18(2):123-36. Epub 2016/06/09. doi:10.1684/epd.2016.0830. Cited in: Pubmed; PMID 27267311.

2. Megarbane A, Al-Ali R, Choucair N, Lek M, Wang E, Ladjimi M, et al. Temple-Baraitser Syndrome and Zimmermann-Laband Syndrome: one clinical entity? BMC Med Genet. 2016 Jun 10;17(1):42. Epub 2016/06/11. doi:10.1186/s12881-016-0304-4. Cited in: Pubmed; PMID 27282200.

3. Bramswig NC, Ockeloen CW, Czeschik JC, van Essen AJ, Pfundt R, Smeitink J, et al. 'Splitting versus lumping': Temple-Baraitser and Zimmermann-Laband Syndromes. Hum Genet. 2015 Oct;134(10):1089-97. Epub 2015/08/13. doi:10.1007/s00439-015-1590-1. Cited in: Pubmed; PMID 26264464.

4. Cappi C, Oliphant ME, Peter Z, Zai G, Conceicao do Rosario M, Sullivan CAW, et al. De Novo Damaging DNA Coding Mutations Are Associated With Obsessive-Compulsive Disorder and Overlap With Tourette's Disorder and Autism. Biol Psychiatry. 2020 Jun 15;87(12):1035-1044. Epub 2019/11/28. doi:10.1016/j.biopsych.2019.09.029. Cited in: Pubmed; PMID 31771860.

5. Satterstrom FK, Kosmicki JA, Wang J, Breen MS, De Rubeis S, An JY, et al. Large-Scale Exome Sequencing Study Implicates Both Developmental and Functional Changes in the Neurobiology of Autism. Cell. 2020 Feb 6;180(3):568-584 e23. Epub 2020/01/26. doi:10.1016/j.cell.2019.12.036. Cited in: Pubmed; PMID 31981491.

6. Gripp KW, Smithson SF, Scurr IJ, Baptista J, Majumdar A, Pierre G, et al. Syndromic disorders caused by gain-of-function variants in KCNH1, KCNK4, and KCNN3-a subgroup of K(+) channelopathies. Eur J Hum Genet. 2021 Sep;29(9):1384-1395. Epub 2021/02/18. doi:10.1038/s41431-021-00818-9. Cited in: Pubmed; PMID 33594261.

7. Wang H, Zhang X, Ding H. Temple-Baraitser syndrome with KCNH1 Asn510Thr: a new case report. Clin Dysmorphol. 2021 Jan;30(1):27-31. Epub 2020/09/22. doi:10.1097/MCD.0000000000000345. Cited in: Pubmed; PMID 32956079.

8. Rossi M, El-Khechen D, Black MH, Farwell Hagman KD, Tang S, Powis Z. Outcomes of Diagnostic Exome Sequencing in Patients With Diagnosed or Suspected Autism Spectrum Disorders. Pediatr Neurol. 2017 May;70:34-43 e2. Epub 2017/03/24. doi:10.1016/j.pediatrneurol.2017.01.033. Cited in: Pubmed; PMID 28330790.
